# The contribution of the intestinal microbiome to immune recovery after HCT

**DOI:** 10.3389/fimmu.2022.988121

**Published:** 2022-08-18

**Authors:** Alex E. Wolfe, Kate A. Markey

**Affiliations:** ^1^ Clinical Research Division, Fred Hutchinson Cancer Center, Seattle, WA, United States; ^2^ Division of Medical Oncology, University of Washington, Seattle, WA, United States

**Keywords:** microbiota, immune reconstitution, allogenic hematopoietic stem cell transplantation, metabolites, graft vs host disease (GVHD)

## Abstract

Allogenic hematopoietic stem-cell transplantation (allo-HCT) is a curative-intent immunotherapy for high-risk hematological malignancies and immune deficiencies. Allo-HCT carries a high risk of treatment-related mortality (TRM), largely due to infection or graft-versus-host disease (GVHD). Robust immune recovery is essential for optimal patient outcomes, given the immunologic graft-versus-leukemia effect prevents relapse, and functional innate and adaptive immunity are both needed for the prevention and control of infection. Most simply, we measure immune recovery by enumerating donor lymphocyte subsets in circulation. In functional terms, ideal immune recovery is more difficult to define, and current lab techniques are limited to the measurement of specific vaccine-responses or mitogens *ex vivo*. Clinically, poor immune function manifests as problematic infection with viral, bacterial and fungal organisms. Furthermore, the ideal recovering immune system is capable of exerting graft-versus-tumor effects to prevent relapse, and does not induce graft-versus-host disease. Large clinical observational studies have linked loss of diversity within the gut microbiome with adverse transplant outcomes including decreased overall survival and increased acute and chronic GVHD. Furthermore, the correlation between intestinal microbial communities and numeric lymphocyte recovery has now been reported using a number of approaches. Large sets of clinically available white blood cell count data, clinical flow cytometry of lymphocyte subsets and bespoke flow cytometry analyses designed to capture microbiota-specific T cells (e.g. Mucosal-associated invariant T cells, subsets of the gd T cells) have all been leveraged in an attempt to understand links between the microbiota and the recovering immune system in HCT patients. Additionally, preclinical studies suggest an immunomodulatory role for bacterial metabolites (including butyrate, secondary bile acids, and indole derivatives from tryptophan metabolism) in transplant outcomes, though further studies are needed to unravel mechanisms relevant to the post-HCT setting. An understanding of mechanistic relationships between the intestinal microbiome and post-transplant outcomes is necessary for reduction of risk associated with transplant, to inform prophylactic procedures, and ensure optimal immune reconstitution without alloreactivity. Here, we summarize the current understanding of the complex relationship between bacterial communities, their individual members, and the metabolites they produce with immune function in both the allo-HCT and steady-state setting.

## Introduction

Allogenic hematopoietic stem-cell transplantation (allo-HCT) is a curative-intent immunotherapy for hematological malignancies and immune disorders, performed for over 25,000 patients annually (1, 2). Successful HCT delivers a robustly reconstituted new donor immune system which is a) capable of exerting graft-versus-tumor effects to prevent relapse, b) minimally alloreactive to minimize Graft-vs-Host Disease (GVHD), c) able to generate adequate responses to control viral, bacterial and fungal infections (3, 4).

The process of allo-HCT involves administration of a cytotoxic conditioning regimen which leads to intensity-dependent damage of the gut epithelium (4, 5), with concurrent translocation of damage and pathogen-associated molecular patterns (DAMPS/PAMPS) into the circulation (4). Following conditioning, infusion of the donor graft and subsequent multilineage repopulation of immune cells are key for successful patient outcomes. Loss of α-diversity of the intestinal microbiota has been associated with worse overall survival (OS) (6, 7), development of infection (7, 8), and acute GVHD (aGVHD) (9) in the early recovery period. Specific taxa have been linked with an increased risk of chronic GVHD (cGVHD) at later timepoints (10).

Acute and chronic GVHD are both products of immune hyperactivation. aGVHD occurs when incoming donor immune cells recognize recipient antigen as foreign and respond in a way that results in tissue damage (5). The terminal effectors of aGVHD are alloantigen-specific T cells, which produce pro-inflammatory cytokines and cause host tissue apoptosis in an uncontrolled positive feedback loop (4, 5). aGVHD pathogenesis has three key stages: Firstly, initial conditioning-related host tissue damage results in the release of DAMPS from host cells as well as the release of bacterial products such as lipopolysaccharide (LPS) into systemic circulation (4). This first stage is followed by production of pro-inflammatory cytokines such as tumor necrosis factor alpha (TNF-α), interleukin (IL)-1β, and IL-6 (4). This pro-inflammatory cytokine milieu, coupled with the recruitment, priming, and expansion of donor T cells, results in the third stage: prolific cytokine production [interferon-gamma (IFN-γ), IL-17] and cytotoxic activity by CD8+ T cell subsets which cause further direct tissue damage (4, 5). In parallel, macrophages, neutrophils, and other innate effector cells are recruited and activated and serve to amplify cytokine production (4). While prior aGVHD is a key risk factor, cGVHD is a late complication with a different pathogenesis, characterized by dysregulated B and T cell immunity, autoantibody production, and a fibrotic phenotype predominantly involving the skin, lung and liver (4, 11, 12).

Optimal immune recovery is critical for successful long-term outcomes for transplant patients. Our understanding of the determinants of immune reconstitution remains poor, however recent data relating to the intestinal microbiome and the recovery of lymphocyte populations suggests that this is a rich area for exploration. There are a number of lines of evidence supporting a link between hematopoietic development and the microbiome:

- Associative clinical studies utilizing large cohorts of clinical data and methodology such as flow cytometry of lymphocyte subsets and microbiome analysis have linked the intestinal microbiome to immune cell recovery post-HCT (13, 14).
- Preclinical studies have demonstrated that the intestinal microbiome may influence hematopoiesis *via* toll-like receptor (TLR) signaling to hematopoietic stem cells (HSCs) (15) and overall numeric immune reconstitution in syngeneic transplant models (16).
- Bacterial metabolites such as butyrate (10), secondary bile acids (17), and indole derivatives through bacterial tryptophan metabolism (18) modulate immunophenotype, though further studies are needed to unravel mechanisms specific to a post-HCT setting.

**Figure 1:**
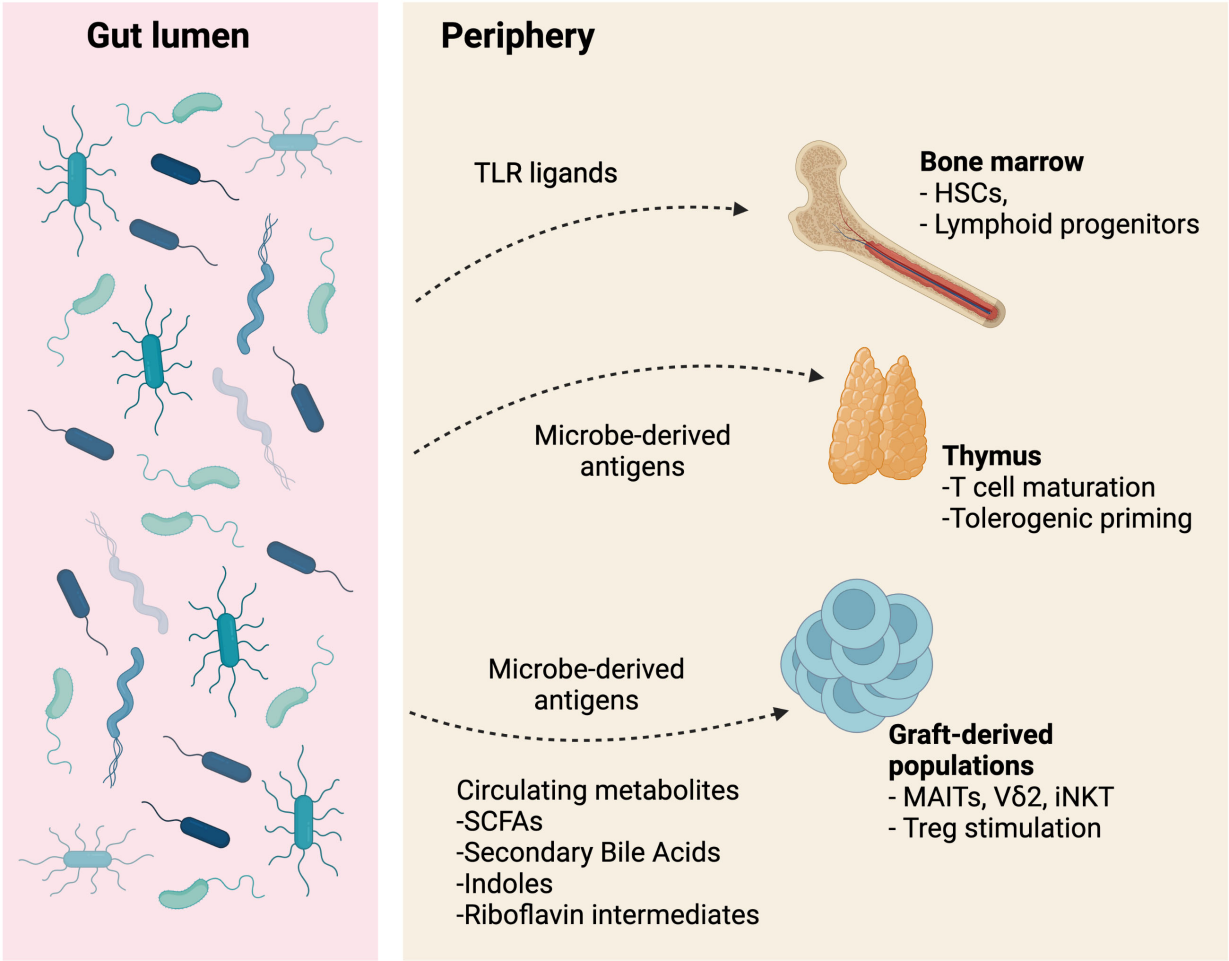
The relationships between intestinal bacteria, bacterial ligands, and bacterial metabolites influence bone marrow, thymic, and graft-derived immune development and maintenance. Image created with BioRender.com.

An understanding of mechanistic relationships between the intestinal microbiome and post-transplant outcomes is invaluable for risk reduction *via* prophylactic procedures with the goal of proper immune reconstitution without alloreactivity. Here, we summarize the current understanding of the complex relationship between bacterial communities, their individual members, and the metabolites they produce with immune function in both the allo-HCT and steady-state setting. **Figure 1** provides a simplified outline of these concepts.

## Associations between the intestinal microbiome, immune reconstitution, and post-transplant outcomes in humans

A properly reconstituted immune system requires both innate and adaptive arms for defense against viral and microbial pathogens, tissue repair, and beneficial graft-vs-tumor effects (4). Immune reconstitution is dependent on factors including conditioning regimen, graft source and manipulation, transplant outcomes such as GVHD, and fixed patient factors including age (19). CD4^+^ and CD8^+^ T lymphocytes in the peripheral blood typically take in the order of 3-6 months post-allo-HCT to recover to near-normal levels (19, 20). This recovery reflects both the proliferation of graft T cells and *de novo* production of mature T cells, which requires bone marrow (BM) development of lymphoid progenitors as well as adequate thymic function (20, 21). However, conditioning-related damage, which can be further exacerbated by aGVHD, impairs thymic function (22). The optimal (or even adequate) CD4+ T cell count after allo-HCT is not perfectly defined, however recovery (to >50 cells/μL) has been associated with improved overall survival in pediatric patients (14), and was a predictor of both non-relapse mortality (NRM) and OS in a cohort of 554 pediatric and adult patients receiving CD34-selected grafts, hereby referred to as T-cell depleted (TCD) grafts (23). Adequate T cell function, as measured by response to T cell mitogens such as phytohemagglutinin, is predictive of OS by six months post-TCD-engraftment, with 38% of patients reaching normal proliferative capacity by 12 months (24). In practical terms, a count of 200 cells/μL in the absence of GVHD or other complications is often used as a threshold for cessation of prophylactic anti-infective medications, though these guidelines are controversial in the context of allo-HCT and often decided on an individual basis (25). With respect to the other lymphocyte subsets, the B cell compartment can take years following transplant to reach normal levels (14, 20). NK cell recovery, in contrast, is usually relatively rapid, with counts typically reaching normal limits within weeks (14, 24). In the setting of T cell replete, or unmodified grafts, immune reconstitution typically occurs more rapidly than when TCD grafts are employed, due to the expansion of mature T cells present in the graft (19).

Several studies have reported associations between both α-diversity and specific bacterial taxa within the gastrointestinal tract with transplant outcomes (6, 7). Alpha-diversity is defined as a measure of richness and evenness of bacterial taxa within a single microbial community or patient sample (26). This is a useful summary measure but does not offer insights into the specific compositional differences that may exist between different samples and patient groups. To understand whether there are differences in microbial community structure, other techniques are required, and include comparison of β-diversity (using PERMANOVA testing), linear discriminant analysis effect size (LefSE) (27) and Bayesian statistical approaches (10, 13, 26). Though data from randomized interventional studies are lacking, the loss of α-diversity can likely be attributed to hospitalization, changes in diet, as well as direct damage from myeloablative conditioning, prophylactic treatment, GVHD, and other antimicrobial therapies (3–5, 7, 13). Low α-diversity has been correlated with infection and GVHD-related deaths following transplantation (6, 7). Patients with peri-neutrophil-engraftment stool α-diversity in the lowest tertile are three times more likely to succumb to non-relapse mortality within three years (7). Mono-domination (>30% relative abundance) of the intestinal microbial community by a single taxon is also a common feature of lower-diversity microbial communities, and has been reported as near-universal during the peri-neutrophil-engraftment period (6). Mono-domination is of interest because, both bacteria (*Enterococcus* spp.) (8, 28) and fungi (*Candida* spp.) (29) have been observed as dominating species preceding blood stream infection. Individual taxa have also been associated with GVHD and OS, as highlighted in **Table 1**.

**Table 1:** Clinical Data Associating Microbial Features with Transplant Outcome.

Study	Patient Numbers	Good Prognosis	Poor Prognosis
Taur et al, 2012 (8)	n=94 allo-HCT patients Fecal samples from pre to d+35	Not reported	Domination by *Enterococcus*, *Streptococcus*, *Proteobacteria* with ↑ bacteremia
Taur et al, 2014 (7)	n=80 allo-HCT patients Fecal samples peri-neutrophil engraftment	↑ *Lachnospiraceae*, *Actinomycetes* with ↑ OS	↑ Gamma-proteobacteria with ↓ OS ↓ α-diversity with ↓ OS Domination by *Enterococcus*, *Streptococcus*, *Enterobacteriaceae*, and *Lactobacillus* spp.
Jenq et al, 2015 (30)	2 cohorts, (n=64 and n=51) Fecal samples collected ~d+12	↑ *Blautia* with ↓ GVHD mortality and ↑ OS	Not reported
Golob et al, 2017 (9)	n=66 allo-HCT recipients Fecal samples at neutrophil recovery n=36 healthy HCT donors	↑ *Bacteroides thetaiotaomicron*, *B. ovatus*, *B. caccae* and *Lachnospiraceae* and *Butyricicoccus* spp. with ↓ severity aGVHD	↑ *B. dorei, Rothia mucilaginosa*, *Solobacterium moorei*, and *Veillonella parvula* with↑ severity aGVHD
Stein-Thoeringer et al, 2019 (28)	n=1,325 allo-HCT recipients Fecal samples peri-transplant	Not reported	Domination by *Enterococcus (E. faecium)* with ↓ OS and ↑ GVHD mortality
Peled et al, 2020 (6)	n=1362 allo-HCT patients Fecal samples pre-transplant and ~weekly post-transplant	↑ α-diversity in transplantation period with ↑ OS	↑ *Enterococcus*, *Klebsiella*, *Escherichia*, *Staphylococcus*, *Streptococcus* spp. in ↓ diversity patients Domination by *Enterococcus* and *Streptococcus* spp. with ↑ GVHD risk
Markey et al, 2020 (10)	n=54 cGVHD and n=171 control allo-HCT patients Fecal samples between d−30 and d+365	d+100 ↑ *Lachnoclostridium*, and *Clostridium* with cGVHD-free	d+100 ↑ *Akkermansia* and *Streptococcus* prior to cGVHD ↓ *Faecalibacterium* prior to cGVHD
Greco et al, 2021 (31)	n=100 allo-HCT patients Fecal samples d-7, 0, + 10, +30	d+10 ↑ *Lachnospiraceae* with reduced aGVHD risk at day +10 post-transplant	d+10 ↑ *Enterococcaceae*, *Staphylococcaceae* associated with aGVHD

↑ (increase); ↓ (decrease).

### Relationship of the microbiota with hematopoietic progenitor populations

There are a number of mouse studies supporting a relationship between microbial signals and hematopoiesis (32–36). Impaired hematopoiesis has been observed in germ-free (GF) mice, and antibiotic treatment has been shown to decrease hematopoiesis. Furthermore, it has been proposed that microbe-derived factors participate in regulation of genes involved in cell lineage commitment (myeloid ecotropic viral integration site 1, hepatic leukemia factor, PR/SET Domain 16 or *meis1*, *hlf*, and *prdm16* respectively) and proliferation (activator protein 1 or *ap1*) *via* a capacity to influence methylation status, though this is less well understood (37).

The GF mouse, devoid of any microbiota, has a marked impairment of hematopoiesis, particularly in cells of myeloid lineage, leading to systemic numeric deficits in innate immune cell populations such as neutrophils, monocytes and macrophages (32). Nucleotide-binding oligomerization domain 1 (NOD1) is an intracellular receptor expressed by innate immune and epithelial cells which is usually responsive to bacterial cell wall fragments (*i.e*. peptidoglycan), which activate the nuclear factor kappa B (NF-κB) pathway to promote an inflammatory and antimicrobial response (38, 39). NF-κB is considered a regulator for innate immunity and is involved in pro-inflammatory pathways (40). Treatment of GF mice with a purified ligands (meso-diaminopimelic acid or muramyl dipeptide, two peptidoglycan components) for NOD1 was sufficient to restore hematopoietic stem cells and progenitors in the BM, as well as serum concentrations of hematopoietic cytokines and growth factors such as IL-7, Fms Related Tyrosine Kinase 3 Ligand (Flt3L), stem cell factor (SCF), and thrombopoietin (ThPO), suggesting that microbe-derived signals have a direct capacity to influence hematopoiesis (33).

In addition to the data from the GF mouse setting, treatment of conventional specific-pathogen-free (SPF) mice with broad-spectrum antibiotics leads to a loss of BM granulocytes (34). Kanamycin [an antibiotic broadly targeting microbial RNA with activity against most Gram-positive and Gram-negative bacteria (41)] was administered to C3H/H3N mice for 1-2 weeks prior to immunophenotyping of immune populations in liver, spleen, thymus, BM, and peripheral blood using flow cytometry (34). Both the proportion and absolute number of granulocytes (Gr-1^+^Mac-1^+^) in the bone marrow and peripheral blood were significantly decreased following kanamycin treatment (34). GF or antibiotic-treated mice display impaired hematopoiesis and myelopoiesis in the bone marrow compared with conventional mice, manifesting as both reduced proportions and total numbers of myeloid and lymphoid progenitors (24, 26). Mice administered an antibiotic cocktail of vancomycin, neomycin, ampicillin, and metronidazole in their drinking water for 2 weeks developed systemic leukopenia and a reduction of BM hematopoiesis (as measured by bromodeoxyuridine proliferation assays of HSC populations *via* flow cytometry), which was partially rescued by fecal microbiota transfer (FMT) (36). A similar immunophenotype was seen in Signal Transducer and Activator of Transcription 1 (Stat1) knockout (KO) mice, suggesting a role for Stat1, a transcription factor involved in the activation of T cells downstream of IFN signaling (36). Specifically, GF or antibiotic-treated Stat1 KO mice display impaired hematopoiesis and myelopoiesis in the bone marrow compared with conventional mice (36). The transfer of either serum collected from SPF mice or 10^4^ CFU of heat-killed *Escherichia coli* to GF mice was able to restore myeloid cell numbers in wildtype (WT) mice, but not MyD88/TICAM double knock-out mice, implicating GM detection downstream of systemic TLR signalling (35). MyD88 (Myeloid Differentiation Primary Response 88) and TICAM (Toll-IL-1-homology domain-containing adaptor molecule 1) are downstream signaling molecules of TLRs expressed on mammalian cells, receptors which activate inflammatory pathways in response to bacterial ligands (42). Briefly, TLR1-7 and 9 are important for the recognition of microbial antigens, including triacylated and diacylated lipoproteins, flagellin, LPS, and viral double-stranded RNA (42). Interestingly, using a syngeneic T-cell depleted bone marrow transplant setting, Staffas and colleagues (16) observed that sucrose administration could reverse severe lymphopenia in antibiotic-treated, irradiated mice, suggesting a role for nutrient processing by the host intestinal microbiota in T cell reconstitution. Sucrose supplementation of these GM-depleted mice, which can be absorbed directly by the host without processing by the microbiota, normalized thymocyte counts and increased bone marrow cellularity and myeloid frequency (16).

Though incompletely understood, the intestinal microbiome has also been implicated in the regulation of gene transcription which impacts the proliferative capacity as well as lineage determination of HSC (37), putatively *via* the ten-eleven translocation (TET) family of proteins. TET are associated with passive and active DNA demethylation for the regulation of gene expression and are thought to be an early mutation in clonal hematopoiesis (CH) in several blood malignancies such as secondary acute myeloid leukemia and myeloproliferative neoplasia (43). Conditional knock-out mice lacking TET2 in only the LSK (Lin^-^Sca-1^+^c-Kit^+^) compartment were observed to have increased replication of hematopoietic stem cells *in vitro* (measured by replating capacity) and aberrant hematopoiesis *in vivo*, manifesting as increases in the LSK (Lin^-^Sca-1^+^c-Kit^+^), granulocyte, and monocyte populations and splenomegaly in mice (44). Another study found that TET2-deficient mice were more susceptible to dissemination of bacteria from the jejunum into the blood, correlated with a downregulation in junction proteins such as zonula occludens-1 (ZO-1) (45). When compared with those from GF mice, stem cells from conventional mice were found to be significantly hypomethylated in regulatory regions called low methylated regions (LMR) which correspond to specific genes, including AP-1 and NF-κB binding sites, and these differences correlated with a significantly higher expression in TET3 levels in conventional mice (46). AP-1, comprised of members of the Jun and Fos protein families, plays a role in cell proliferation, differentiation, inflammation, and migration. Additionally, AP-1 regulates expression of MyD88, a molecule downstream of bacterial-sensing TLR4 (46). Additionally, AP1 is required for TCR signaling and cytokine production indispensable for immune regulation, and levels of c-Fos expression were correlated with long-term survival of allo-HCT patients vs those who died within three years post-transplant (47). Another group studied 4.6 million methylated sites in multipotent progenitors and downstream myeloid and lymphoid progenitors, to better understand how epigenetic modification influenced immune system development (37). They observed an inverse relationship between differential DNA methylation and subsequent gene expression, with lymphoid rather than myeloid commitment more dependent on DNA methylation (37).

Very little is known about which specific bacterial taxa may influence stem cell differentiation, but experiments involving different combinations of antibiotics, colonization of mice with specific bacterial communities of interest, or *in vitro* approaches exploring specific effects of bacterial products on cell lines of interest are likely to offer useful insights.

### Lymphoid progenitors

Lymphoid and myeloid progenitors differentiate from hematopoietic stem cells through a complex series of cues, regulation of transcription factors, and epigenetic modifications (32). Two clinical studies have laid important groundwork for establishing the relationship between the microbiome and immune recovery after allo-HCT in humans. In their recent study, Schluter and colleagues (13) analyzed clinical white blood counts (WBC) in a small cohort of patients who received autologous fecal microbiota transfers (auto-FMT; n = 14) at the time of neutrophil engraftment (>500 neutrophils/μL for three consecutive days) as part of an interventional clinical trial conducted originally by Taur et al. (48) In the primary study, Taur et al. explored the capacity of auto-FMT (n=14 auto-FMT, n=11 control) to replenish intestinal microbiota diversity following transplant and associated antibiotic-induced disruption. They report a significant improvement in the alpha-diversity of stool samples from patients who received auto-FMT ~18 days post-transplant up until study end 1 year later (48). Schluter et al (13) found that, during the first 100 days post-neutrophil engraftment, the total WBC was increased compared with controls. The authors went on to match daily blood count data with densely collected stool samples to link microbial community changes with subsequent shifts in the daily blood counts (13). To explore the hypothesis that peri-neutrophil-engraftment features of the microbiome are linked with overall outcome (as discussed above) due to a relationship with immune recovery, the Miltiadous study (14) analyzed stool samples from the d7 to 21 window and related them to immunophenotyping data obtained *via* flow cytometry analyses at d100 post-transplant. The study designs and key findings of these two studies are highlighted below in **Table 2**.

**Table 2:** Clinical Associations between Intestinal Microbiota and Immune Reconstitution.

Study Name	Patients	Data Summary	Positive Associations	Negative Associations
Schluter et al, 2020 (13)	a) n=24 auto-FMT allo-HCT patients b) n=841 allo-HCT patients	a) Daily Complete Blood Count and lymphocyte count, 3 year OS b) Fecal microbiome data and early (d0-d100) clinical CBC data	↑ *Faecalibacterium*, *Ruminococcus*, *Akkermansia* with ↑ neutrophil rates ↑ *Ruminococcus 2* and *Staphylococcus* with ↑ lymphocyte counts ↑ *Ruminococcus 2* and *Faecalibacterium* with ↑ monocyte rates of change	↑ *Rothia* and *Clostridium sensu strictu 1* with ↓ neutrophil rates ↑ *Clostridium sensu strictu 1* with ↓ monocyte rates
Miltiadous et al, 2022 (14)	n=894 allo-HCT patients	Peritransplant fecal microbiome data and late (d100) immunophenotyping	↑ *Erysipelotrichaceae* with > median CD4 recovery in PBSC grafts ↑ α-diversity with ↑ CD4 and CD8 counts	↑ *Enterobacteriaceae*, *Enterococcus*, and *Staphylococcus* with < median CD4 recovery ↑ *Bacillus* with ↑ CD4 recovery No relationship of α-diversity with B or NK counts

↑ (increase); ↓ (decrease).

### Thymic selection and expansion

Long term immune reconstitution relies on the *de novo* generation within the BM and subsequent selection of a diverse repertoire of T cells in the thymus. The thymus is necessary for the selection of a diverse T cell repertoire (21). Thymic tissue, already less functional in adults than in young children, is damaged by pre-transplant conditioning and is exacerbated if GVHD develops (21). Though thymopoiesis is often detected from 6 to 8 months post-transplant (using a PCR assay on PBMC samples to detect T-cell receptor excision circles (TRECs) as a measure of thymic functionality), delayed or impaired regeneration of the thymus in umbilical cord blood transplant patients leads to lymphopenia and thus poor functional T cell recovery with poor overall prognosis (22).

A recent study in mice has explored microbiota-specific education of T cells in the thymus upon neonatal and adult colonization (49). The authors reported that commensal-dependent thymic expansion occurs at steady-state, by comparing Segmented Filamentous Bacteria (SFB)-specific CD4+ T cells in the thymi of adult and weanling mice two weeks post-colonization with SFB using enrichment *via* magnetic SFB3340 tetramers (49). SFB colonization led to increases in circulating T_H_17^+^ cells in adult mice, consistent with observations from previous studies (50–53). They also observed increased thymic expansion of SFB-specific CD4+ T cells in weanling mice, and an increase in total bacterial DNA in the thymus and mesenteric lymph nodes (mLNs) of young but not adult mice (49). The difference in migration of bacterial DNA between adult and weanling mice was confirmed to require CX3CR1+ dendritic cells (DC) present at a narrow developmental window in weanling mice, and this thymic priming with SFB and other bacteria such as *E. coli* was protective in cases of cross-reactive pathogens or overgrowth of pathobionts (49). These data suggests there may be a limited time window for thymic presentation of microbiota-derived antigens, which may be relevant after allo-HCT during the phase of *de novo* T cell development.

While some SFB research has been performed in humans, SFB is often below the limit of detection in adults. In mice, it is believed that the specific attachment of SFB to the small intestinal epithelial cells (ECs) is responsible for this T_H_17 induction, as a rat-specific strain of SFB, which could not adhere to mouse ECs, could not induce T_H_17 in a murine model, but the EC-adherent *Citrobacter rodentium* and *E. coli* 0157 did elicit T_H_17 responses (50). Thus, this gives merit to the exploration of not only SFB but adherent SFB equivalents in a human clinical context as potent, specific immunomodulators (54).

### Maintenance of mature microbiota-dependent populations

A number of microbe-dependent cell types have now been studied in the context of allogeneic stem cell transplantation. Mucosal-associated invariant T cells (MAIT) respond to bacteria and fungi through detection of riboflavin metabolites presented in the context of a class I-related molecule known as MR1 (3). MAIT development is dependent on a very narrow window of exposure to ligands produced by riboflavin-synthesizing bacteria within the first few weeks of life in humans, and in GF mice these cells fail to develop (55). Preclinical and clinical studies have examined the role of MAIT cells in transplant outcome and their relationship with the gut microbiome.

In the mouse, MAIT cells are predominantly found in the tissues, particularly in the liver, lung, and the lamina propria in the colon and small intestine (56). MAIT cells are best-defined using an MR1-tetramer loaded with activating ligand 5-OP-RU, in conjunction with CD3, CD4, and CD8 and, in humans, CD161 and Vα7.2 (56, 57). In mouse models of allo-HCT, recipient MAIT cells appear to be protective against aGVHD, with MR1 deficient animals experiencing more rapid lethal GVHD than their WT counterparts (56). This is thought to be due to IL-17-dependent effects on antigen presentation and intestinal barrier integrity (56). As MAIT cells are microbiota dependent, these mouse findings may offer a partial explanation for the poor outcome seen in patients with pre-transplant dysbiosis (6).

In humans, MAIT cells represent up to 10% of all circulating CD3+ T cells (3). In the setting of clinical allo-HCT, frequency of donor graft-derived MAIT cells in the early post-HCT period has been linked with favorable transplant outcomes in a number of studies (58, 59). We have recently reported a relationship between MAIT cells and transplant outcome, in a cohort of 118 PBSC graft recipients (59). A higher than median MAIT cell frequency at day 30 post-transplant was correlated with OS and decreased NRM. An association between MAIT frequency and lowered incidence of grades 2 to 4 aGVHD as well as GI GVHD was also seen (59). Prior to our study, Bhattacharyya et al (58) had observed that increases in absolute MAIT cell count (in increments as small as 10 MAIT cells/μL) in the blood in the early post-HCT period were associated with a reduced risk of GVHD in a cohort of 105 patients. Lower MAIT cell counts were associated with development of more severe grades of aGVHD (58). The latter study confirmed the circulating MAITS by day 100 post-transplant to be almost exclusively graft-derived, and this reconstitution occurs early but rapidly before plateauing from day 30 to 100 post-transplant (58). Interestingly, a study investigating cord blood transplant recipients reported that increased MAIT numbers were linked with mild cGVHD compared to those without GVHD (n = 173 patients >1 year from transplant with matched sibling donor or umbilical cord blood) (60).

A correlation between α-diversity and MAIT proportion and number in circulation at d+30 post-transplant has been observed (59). MAIT frequency has also been positively correlated with relative abundance of members of the classes *Bacteroidia* and *Erysipelotrichia* as well as the family *Ruminococcaceae* and genus *Blautia* (59). *Blautia* and *Bifidobacterium* spp. enrichment was found positively correlated with MAIT cell total counts within 100 days post-PBSC engraftment (58). In a further limited study (n=12), the genera *Blautia*, *Lachnoclostridium*, and *Faecalibacterium* correlated with patients with high MAIT counts, while *Enterococcus*, *Streptococcus*, and *Lactobacillus* spp. correlated with patients with low MAIT counts (n=10) (61).

The microbial analysis highlights that a diverse microbiome may be linked with good transplant outcomes because of its capacity to support protective cell populations, including MAIT cells. Surprisingly for an apparently regulatory population, single-cell RNA sequencing of purified MAIT cells from 5 allo-HCT patients revealed an upregulation of genes associated with effector function and cytotoxicity (granulysin, perforin 1, and C-C motif chemokine ligand 4 or *GNLY, PRF1*, and *CCL4* respectively) and migration (adhesion molecules integrin beta-2 and CD74) compared with healthy controls (59). Purified MAIT cells from HCT patients also demonstrated transcriptional downregulation of negative feedback inhibitors (*NFKBIA* and *TNFAIP3* or TNF alpha induced protein 3) of the NF-κB pathway (59). In terms of functional assays, post-HCT MAITs have the capacity to suppress CD4+ T cell proliferation *in vitro*, but the *in vivo* mechanisms for this remain unclear *(* 58 *).*

Gamma Delta (γδ) T cells have also been implicated in transplant outcomes. In humans, γδ T cells comprise 0.5-10% of circulating CD3+T cells (62) and upon recognition of microbial-derived phosphoantigens, produce perforin, granzyme, and proinflammatory cytokines IFNγ and tumor necrosis factor alpha (TNFα) (3). Like MAIT cells, the Vdelta2 (Vδ2) subset of γδ T cells (of which there is no murine analog) was linked with lower rates of aGVHD (59). These Vδ2 cells were associated with higher than median peri-engraftment stool α-diversity and demonstrated upregulation of GNLY, PRF1, CCL4 and Granzymes B and H compared with controls (59). In contrast to our findings, in their cohort of 105 patients, Gabella et al (63) observed that higher graft frequencies of CD8+ γδ and CD8+Vδ2 cells were positively associated with the development of grades II and III aGVHD but not cGVHD. These contrasting findings highlight that there is more work to be done to understand the role of this population after allo-HCT.

One study noted that, in WT steady-state mice, γδ cells in the intraepithelial lymphocyte (IEL) fraction of the small and large intestines was equivalent between GF and SPF mice, and were not altered through the conventionalization process (64). Another noted that antibiotic-induced dysbiosis (via a 2 week treatment of amoxicillin and clavulanic acid) in an ischemic model led to decreased numbers of IL-17+ γδ T cells in the lamina propria (LP) of the small intestine, but not the colonic LP, peripheral blood, lymph nodes, or spleen (65). In terms of cancer outcomes, Jin et al (66) found that GF mice displayed reduced tumor cell proliferation, coupled with lower frequencies of IL-17+ γδ T cells, a phenomenon which was reversed with conventionalization.

Thus far, there does not seem to be a direct link between the intestinal microbiota and systemic Invariant Natural Killer T cell (iNKT) expansion and development (4). iNKT are another important unconventional T cell subset, which recognize α-galactosylceramide and other glycosphingolipids in the context of the MHC-class I-like molecule, CD1d (3, 67). The reconstitution of iNKT, specifically the human Vα24+NKT, is heavily dependent on graft source, with rapid normalization of this population in recipients of PBSC grafts, but lengthy reconstitution times (>1 year) in recipients of bone marrow grafts (67). Lower Vα24+NKT numbers were correlated with higher incidence of chronic GVHD by 150 days post-transplant and, in BMT-recipients specifically, a reduced incidence of aGVHD (67). Because iNKT cells act as immunomodulators of conventional T cells, Rubio et al (68) investigated iNKT as a ratio to conventional T cells in 71 allo-HCT patients, finding a higher ratio (>10^-3^) associated with absence of aGVHD as well as improved OS and lower NRM, with day 15 ratios predictive of aGVHD risk. In a cohort of 117 allo-HCT patients, the absolute numbers of graft iNKT cells in combination with the *ex-vivo* expansion capacity of iNKT was higher in recipients of PBSC grafts. Patients with fewer iNKTs with less expansion capacity appeared to experience higher grades of GVHD (II-IV) (69). The protective capacity of iNKT T cells against GVHD is thought to be *via* an IL-4 dependent influence on forkhead box P3 (FoxP3)+T_reg_ cells (70). Interestingly, the ligand for iNKT is produced by certain soil-dwelling bacteria as well as *Bacteroides fragilis*, which resides in the gut. Mono-colonization with *B. fragilis* or exogenous, purified *B.* fragilis glycosphingolipids was sufficient to inhibit iNKT cell proliferation, but only in the colon and only within a critical early window (prenatal stage in mice) (71).

## Putative mechanisms by which the intestinal microbiome modulates immune reconstitution and post-transplant complications

### Bacterial stimulation of specific T cell populations

The species within the gut microbiome, in concert, contribute genetic diversity and functional flexibility beyond the host genome itself and links environmental factors (e.g. dietary and drug exposure) with host physiology (72). While associative studies have laid important groundwork, we lack mechanistic insight into how bacterial diversity, individual bacterial community members, and community function interact with immune reconstitution and transplant outcome overall. Moreover, much of our understanding of immune function is built on observations in GF and antibiotic-treated mice and relies on a small number of bacterial species which, when introduced into a GF mouse, contribute substantially to the restoration of one or more component of impaired immune development.

Segmented Filamentous Bacteria (SFB) is one of the most well-studied immunomodulatory species. SFB was found to induce T_H_17 cell differentiation following mono-colonization of GF mice (50–52), and its absence is cited as responsible for a scarcity of IL-17-producing CD4+ T cells in mice from Jackson Laboratories compared with those obtained from Taconic Farms (53), which is thought to contribute to an exacerbation of T_H_17-driven rheumatoid arthritis models (73). SFB (though not commonly detected in human microbiome studies) have been detected at low relative abundance as well as correlated with total secretory IgA concentration in the human terminal ilea and upregulated mRNA from T_H_17-related pathways (54).

The direct stimulation of pattern recognition receptors (PRR), such as the aforementioned TLRs and NOD receptors, plays a role in the cross-talk of the intestinal microbiota and adaptive immunity (74). As previously discussed, the NOD1 receptor expressed by innate immune and epithelial cells responds to bacterial cell wall fragments such as peptidoglycan, which can be derived from both gram negative and positive bacteria (38, 39), and TLRs recognize a variety of microbial-derived antigens (42). The interaction of these bacteria with such receptors have already been implicated in the cellularity and hematopoiesis of stem cells and progenitors in the BM, but the impact of specific bacteria on the immunophenotype may vary by species. For example, host recognition of the polysaccharide A (PSA) antigen of *Bacteriodes fragilis* has been associated with restored lymphocyte zone infrastructure in the spleens of WT mice at steady-state (75). When administered to GF mice, *B. fragilis* altered the ratio of T_H_1 to T_H_2 cells (in favor of T_H_2) (75). *B. fragilis* was also found to aid in maturation of DCs *in vitro via* upregulation of major histocompatibility complex II (MHCII) and increasing costimulatory molecules such as CD80 and CD86 in a dose dependent manner (75). Identifying other specific immunomodulatory bacteria which induce robust immune responses will improve our understanding of how microbial communities may skew the immune system—and how these phenotypes may be deleterious or advantageous in a transplant and immune reconstitution setting.

### Circulating metabolites

While no direct relationship between bacterial-derived metabolites and numeric immune reconstitution has yet been established, metabolites such as short-chain-fatty acids (SCFA) (10), secondary bile acids (17), and indole derivatives through bacterial tryptophan metabolism (18) have been implicated in modulating immune function both at steady-state and in a post-transplant setting. Given that immune reconstitution is adversely influenced by the cytokine storm that can occur early after HCT and in the setting of GVHD, it is likely that the immunoregulatory metabolites contribute to immune fitness and overall numeric recovery, at least of some subsets, though data is currently lacking.

#### Short chain fatty acids

SCFAs (fatty acids containing up to 6 carbon atoms, including butyrate, propionate, acetate, and hexanoate) are the byproducts of anaerobic fermentation of dietary fiber. Butyrate specifically provides an important energy source for colonocytes (76) and may help maintain an epithelial hypoxia ideal for anaerobic commensals, as the reduction process absorbs considerable amounts of local oxygen (77). Of note, butyrate, and to some degree some of the other SCFAs, is also a histone deacetylase inhibitor at steady-state, which promotes the upregulation and skewing toward a tolerogenic T_reg_ phenotype that is thought to promote tolerance to commensal bacteria and help quell inflammation in some colitis models (77–79). SCFAs have been implicated in modulating GVHD severity in both preclinical and clinical studies.

The binding of butyrate and propionate to the G protein-coupled receptor 43 (GPR43) receptor on intraepithelial cells (IECs) alone was sufficient to reduce intestinal permeability and alleviate GVHD in one preclinical murine model (80). Butyrate has also been shown to regulate epithelial permeability and integrity by increasing mucin production and inducing Reg3γ (regenerating islet-derived protein 3) and β-defensin production by mouse IECs (77). Treatment of colonocytes directly with butyrate helped reverse radiation-induced enteropathy as well as intestinal erosion by *Salmonella Typhimurium (* 81 *).* Because GPR43 receptor is most specific for propionate and acetate, a further study examined the role of the butyrate-specific GPR109A receptor in the context of mouse allo-HCT, though of note, GPR109A is also a receptor for niacin (82). Contrary to other reports in the literature, which suggest that butyrate is protective after allo-HCT, T cells lacking the GPR109A receptor (which therefore cannot signal in response to butyrate or niacin) induced less GVHD after transplant than littermate controls, though this effect hasn’t been confirmed as butyrate-dependent (82). Increased butyrate production, as a result of a high fiber diet, was shown to increase CD8+ memory T cell response in an Herpes Simplex Virus 1 (HSV-1) transgenic T cell model, resulting in a higher number of effector CD8+ T cells following secondary exposure to their cognate antigen (83).

In one clinical study, an enrichment of genes related to SCFA metabolism corresponded with a lower plasma concentration of butyrate and propionate at day 100 post-transplant in patients who later developed chronic GVHD (10). Bacteria such as *Erysipelotrichaceae* and *Blautia* spp., known to be associated with above median CD4+ recovery and favorable outcomes, are butyrate producers and are also involved in the conversion of primary to secondary bile acids within the gut (discussed below) (14). The precise mechanistic role of butyrate after transplantation remains controversial, with another study (n= 210 HCT recipients with paired weekly stool specimens) suggesting an association between the presence of butyrate producers and more severe GVHD (84). The presence and relative abundance of at least 1 ‘butyrogenic bacteria’ was linked with increased risk of steroid-refractory (n=17 out of 27) or chronic GVHD involving the gut (n=4) following onset of severe acute GVHD, though these observations are limited by small sample size (84).

#### Bile acids

Bile acids, produced by the liver to aid in the digestion of dietary fat, are further converted into secondary bile acids by the intestinal microbiome, resulting in a large pool of bioactive molecules (85). The most prevalent primaries in humans are cholic acid (CA) and chenodeoxycholic acid (CDCA) (86), which are converted into the predominant secondaries lithocholic acid (LCA), ursodeoxycholic acid (UDCA) and deoxycholic acid (DCA), by microbial enzymes (87, 88). The secondary bile acids are immunologically active because they can signal to the farnesoid X receptor (FXR) and G-Protein Bile Acid-activated Receptor (GPBAR) among others, which are expressed by myeloid-derived cells (monocytes and macrophages primarily) in the entero-hepatic system (88).

At steady-state in the mouse, two derivatives of lithocholic acid (LCA), 3-oxoLCA and isoalloLCA have been observed to modulate the T_H_17/T_reg_ axis. 3-oxoLCA inhibited T_H_17 differentiation by direct binding to RORγt (RAR-related orphan receptor gamma) and isoalloLCA increased FOXP3 expression to induce T_reg_ differentiation (85). Exogenous lithocholic/3-oxo-lithocholic acid combination was found sufficient to restore RORγt+ and FOXP3+ T_reg_ populations lost after 4 weeks on a nutrient-depleted diet (89). A diet containing 1% (w/w) exogenous 3-oxoLCA was adequate to suppress SFB-induced T_H_17 differentiation as well as T_H_17 levels in gut inflammatory conditions (85). The pool of bile acids was reported depleted following allo-HCT, but the exogenous supplementation of secondary bile acid tauroursodeoxycholic acid (TUDCA) ameliorated acute GVHD severity through the reduction of antigen presentation by non-hematopoietic cells, upregulation of antimicrobial molecules and intestinal stem cell markers in both organoid and cell-line cultures, and the reduction of serum TNF cytokine levels in two separate murine models (90). Another group found that the reduction of IL-1b and IL-18 occurred in a dose-dependent manner, inhibiting NLRP3 (NOD-, LRR- and pyrin domain-containing protein 3) inflammasome activation in response of LPS stimulation (91).

In humans, metabolomic profiling of samples taken from two cohorts of allo-HSCT patients paired with healthy sibling donors examined metabolite concentrations after transplantation and at the onset of aGVHD (17). Fatty acids, mono and diacylglycerol and primary bile acids were elevated in all transplant patients as compared with healthy controls, but primary and secondary bile acids were significantly increased at the onset of aGVHD as compared to transplant recipients without GVHD, though the specific mechanism has not yet been investigated (17).

#### Tryptophan metabolites

Tryptophan (TRP) catabolism is largely undertaken by the first and rate-limiting enzyme indoleamine 2,3-dioxygenase (IDO) which breaks TRP into kynurenine (KYN) (92). IDO is widely-expressed by intestinal cells, DCs, and endothelial cells (92). Ninety-five percent of KYN is further degraded into a host of secondary metabolites, including kynurenic acid (KYNA), quinolinic acid (QA), picolinic acid (PA), and 3-hydroxykeurenine (3-HK), by the gut microbiota, while the other four to six percent are metabolized into indole, indicant, tryptamine, and skatole (92, 93). Secondary TRP metabolites have been shown to interact with the ubiquitously expressed aryl hydrocarbon receptor (AhR). AhR promotes secretion of barrier-enhancing cytokines and products such as IL-22 (94) and mucin2 (Muc2) (95) and participates in immune response, tumor promotion, detection of environmental pollutants, cell division (96) and T_reg_ induction (97). The production of the majority of these downstream TRP metabolites require cooperation between multiple bacteria, thus the resulting mixture of AhR ligands in the gut lumen and in circulation is heavily influenced by the intestinal microbiome community and the combination of catalytic enzymes which are present (92).

Studies exploring the relationship between TRP, TRP metabolites, and IDO with transplant outcome in both clinical and preclinical studies have had variable results. One clinical study, comparing high-throughput metabolomics between human leukocyte antigen (HLA)-identical HCT recipients and their healthy sibling donors, found an increase in TRP metabolites, microbiota-derived indole compounds, and plasmalogens associated with acute GVHD onset, with an enrichment of complex lipid metabolites and primary bile acids in those which did not develop GVHD (17). Another clinical study similarly found that plasma concentrations of IDO and downstream metabolites KYN and QA are correlated with severity of GVHD (93).

Most mechanistic work with positive associations between TRP metabolism and outcomes has been completed in preclinical transplant models and *in vitro*. IDO, found to be induced by IFN-γ in epithelial cells, antigen presenting cells (APCs) and endothelial cells, has been implicated in the suppression of allograft rejection in small bowel (98), skin graft (99), and cardiac (100) transplants. IDO is thought to suppress GVHD lethality, potentially by suppressing T_H_1 differentiation in favor of CD4+FoxP3+ T_REG_ stimulation (100, 101) which could also lead to a more tolerogenic state against apoptotic cells resulting from conditioning-related damage (102–104). This is supported by the reversal of an immunosuppressive phenotype in BM-derived immature DCs when IDO was depleted, resulting in up-regulated IL-2 and IFN-γ and downregulated IL-4 and IL-10 (105). One putative mechanism is the indirect inhibition of the amino acid-sensitive mTOR (mammalian target of rapamycin) pathway due to IDO-dependent depletion of available TRP in the gut, which promotes transforming growth factor beta (TGF-β)-dependent inhibition of IFN-γ driven inflammatory Ly6c+CD103+ DC priming to further reduce a pro-inflammatory environment detrimental to transplant outcome (106). This interaction between mTOR and IDO might also explain why dietary tryptophan in mice exacerbated chemically-induced inflammation in mouse colitis models (92).

Exogenous addition of indole-3-carboxaldehyde (ICA), an indole derivative, was shown to extend survival in lethally irradiated mice in a model of allo-BMT in a dose-dependent manner, with a reduction in translocation of bacteria to the mLN, indicating improved barrier integrity (18). Inflammatory cytokines such as TNF-α, IFN-γ, and IL-6 were also reduced in gut homogenates (18). KYN has also demonstrated direct immunomodulatory effects through the inhibition of effector T cell proliferation, which has suppressed acute rejection in allograft transplant models (100), and KYN, 3-HK, and 3-hydroxyanthranilic acid also inhibited T cell response in a mixed lymphocyte reaction of T cells and DCs (99). It is likely that the IL-22 induction by TRP and TRP metabolites is partially responsible for the decrease of GVHD severity, as this cytokine not only enhances barrier stability *via* tight junctions and adherents junctions (107, 108), but induces expression of antimicrobial molecules Reg3γ and Reg3β to further protect against bacterial penetration and overgrowth (109).This has the net effect of regulating intestinal homeostasis and preventing the activation of host responses against bacterial tissue and subsequent escalation of inflammatory responses in an already inflammatory environment post-irradiation (107). This, and the inhibition of pro-inflammatory effector phenotypes in T cells, make TRP, its metabolites, and the bacteria which produce interesting candidates as post-transplant immunomodulators.

### Further insights: Where do we go from here?

Numerous associations drawn from preclinical and clinical associative studies have laid the groundwork for future investigations on the effects of specific bacterial taxa, their functional niches, and their interactions with the post-HCT developing immune system. Steps must be taken to understand this on the mechanistic level, as well as to separate causal relationships from associations. In addition, it is unlikely that single taxa act alone, and the entire community may impact the availability and function of different dietary compounds and metabolites, as is commonly accepted to be the case with respect to tryptophan metabolite production. It is also possible that, as is the case for neonatal development of immune populations, there are key windows after allo-HCT where specific bacterial ligands must be present for optimal immune development. This may be the case for MAIT and Vδ2 cells, for example, as well as other populations which are still being studied.

The variability of the human microbiome, which is dependent on geography, diet, drug treatment, and genetics, is a significant confounding factor in clinical studies (110). Preclinical work is invaluable toward unraveling interactions between host and the intestinal microbiome, but efforts must be made to ensure translatability to humans, using multiple orthogonal methods to verify associations and mechanistic phenomena across species. One avenue toward more faithfully recapitulating an adult human immune function and phenotype may be utilizing wild “dirty mice” in addition to SPF mouse studies (111). SPF mice have been shown to more closely resemble a neonatal than adult human immunoprofile, particularly in the effector and effector memory CD4+ and CD8+ T cell compartments (111). “Dirty mice” or even “humanized” mice may add the layer of complexity and diversity both in intestinal microbiome composition and immunophenotype that can better represent numerous combinations of functional niches and players, as seen in the wide interindividual variability of humans.

Overall, it is likely that the loss of α-diversity following transplantation, and resultant alteration in metabolites and antigens available in the gut lumen and in the circulation, has important implications for numeric recovery of immune populations as well as optimal immune function. Further clinical studies and careful mechanistic studies in mice, paired with rationally-designed clinical trials will be key to moving microbiota-targeted therapies forward.

## Author contributions

AW researched and wrote the review, under the guidance and expertise of KM. All authors contributed to the article and approved the submitted version.

## Conflict of interest

The authors declare that the research was conducted in the absence of any commercial or financial relationships that could be construed as a potential conflict of interest.

## Publisher’s note

All claims expressed in this article are solely those of the authors and do not necessarily represent those of their affiliated organizations, or those of the publisher, the editors and the reviewers. Any product that may be evaluated in this article, or claim that may be made by its manufacturer, is not guaranteed or endorsed by the publisher.
